# ‘What gets measured gets managed’: revisiting the indicators for maternal and newborn health programmes

**DOI:** 10.1186/s12978-018-0465-z

**Published:** 2018-02-02

**Authors:** A. C. Moran, A. B. Moller, D. Chou, A. Morgan, S. El Arifeen, C. Hanson, L. Say, T. Diaz, I. Askew, A. Costello

**Affiliations:** 1 World Health Organization, Geneva, Switzerland; 2 University of Melbourne, Melbourne, Australia; 3 ICDDR, B, Dhaka, Bangladesh; 4 Karolinska Institutet, Stockholm, Sweden

**Keywords:** Maternal, Newborn, Measurement, Monitoring

## Abstract

**Background:**

The health of women and children are critical for global development. The Sustainable Development Goals (SDG) agenda and the Global Strategy for Women’s, Children’s, and Adolescent’s Health 2016–2030 aim to reduce maternal and newborn deaths, disability, and enhancement of well-being. However, information and data on measuring countries’ progress are limited given the variety of methodological challenges of measuring care around the time of birth, when most maternal and neonatal deaths and morbidities occur.

**Main body:**

In 2015, the World Health Organization launched Mother and Newborn Information for Tracking Outcomes and Results (MoNITOR), a technical advisory group to WHO. MoNITOR comprises 14 independent global experts from a variety of disciplines selected in a competitive process for their technical expertise and regional representation. MoNITOR will provide technical guidance to WHO to ensure harmonized guidance, messages, and tools so that countries can collect useful data to track progress toward achieving the Sustainable Development Goals.

**Short conclusion:**

Ultimately, MoNITOR will provide technical guidance to WHO to ensure harmonized guidance, messages, and tools so that countries can collect useful data to track progress toward achieving the Sustainable Development Goals.

## Background

The health of women and children are critical for global development. The Sustainable Development Goals (SDG) agenda [1] and the Global Strategy for Women’s, Children’s, and Adolescent’s Health 2016–2030 aim to reduce maternal and newborn deaths, disability, and enhancement of well-being [2]. However, information and data on measuring countries’ progress are limited given the variety of methodological challenges of measuring care around the time of birth, when most maternal and neonatal deaths and morbidities occur. National and global monitoring efforts focus on coverage of antenatal care, skilled attendance during birth and postnatal care, however the content and quality of care during these interactions are largely unmeasured and unreported [3–5]. To fill this gap, several measurement groups are working on indicators and methodologies, providing often conflicting recommendations of what should be measured at global, national and sub-national levels. Their work advances the field, but they confuse countries on what and how to measure or to track progress toward ambitious SDG targets. Although SDG core indicators are agreed, an operational framework and core set of common indicators for all countries is not yet established.

### Main text

The Millennium Development Goals final report stated that “data are an indispensable element of the development agenda” [6]. Other health areas have profited from coordinated measurement initiatives. The Malaria Monitoring and Evaluation Reference group and UNAIDS Monitoring and Evaluation Reference Group facilitate partner alignment, harmonize indicators, and communicate key measurement issues [7, 8].

In 2015, the World Health Organization launched Mother and Newborn Information for Tracking Outcomes and Results (MoNITOR), a technical advisory group to WHO. MoNITOR comprises 14 independent global experts from a variety of disciplines selected in a competitive process for their technical expertise and regional representation. The vision of MoNITOR is to facilitate measurement, align initiatives, and provide technical guidance to WHO. WHO leads by coordinating metrics related to maternal and newborn health.

MoNITOR held two meetings to review ongoing measurement efforts, map maternal and newborn indicators and data sources, and assess gaps. Three co-chairs, technical experts from Australia, Bangladesh and Sweden, advise MoNITOR’s work. Emerging issues include a lack of consistent definitions for key indicators across varying data collection platforms. Over the next 2 years, MoNITOR will provide recommendations for priority indicators, norms and standards for data collection platforms, a coordinated research agenda, and how to build regional capacity. WHO is also building a simpler, real-time, integrated database for maternal and newborn indicators included in the Global Strategy.

## Conclusion

Ultimately, MoNITOR will provide technical guidance to WHO to ensure harmonized guidance, messages, and tools so that countries can collect useful data to track progress toward achieving the Sustainable Development Goals. See http://www.who.int/maternal_child_adolescent/epidemiology/monitor/en/ for additional information.

## Abbreviations

MoNITOR: Mother and Newborn Information for Tracking Outcomes and Results
SDGs: Sustainable Development Goals
